# Pseudotumoral and Multiple Retinal Pigment Epithelium Proliferation in Vogt-Koyanagi-Harada Disease

**DOI:** 10.1155/2015/153831

**Published:** 2015-10-05

**Authors:** Juan B. Yepez, Felipe Murati, Michele Petitto, J. Fernando Arevalo

**Affiliations:** ^1^Vitreoretinal Surgery Department, Clinica de Ojos, Maracaibo, Venezuela; ^2^Retina Division, Wilmer Eye Institute, School of Medicine, Johns Hopkins University, Baltimore, MD 21287, USA

## Abstract

We report a case of pseudotumoral retinal pigment epithelium (RPE) proliferation in Vogt-Koyanagi-Harada (VKH) disease, in a 50-year-old female who presented with a juxtapapillary and peripheral subretinal hyperpigmented lesions in the left eye and “sunset glow fundus,” hyperpigmented striae, and multiple atrophic chorioretinal spots in the periphery. The darkly pigmented exuberant larger subretinal mass extended to the periphery with associated subretinal fibrosis. This patient demonstrated the entire clinical presentation of VKH disease, which tends to course with a chronic, bilateral, granulomatous panuveitis and exudative retinal detachment associated with poliosis, vitiligo, alopecia, and central nervous system and auditory signs. Our case is unique for the presence of exuberant, pseudotumoral RPE proliferation at the juxtapapillary region and peripheral area. Although this complication has rarely been reported, a high index of suspicion is warranted for early diagnosis and avoids unnecessary treatments of a pseudotumor.

## 1. Introduction

Vogt-Koyanagi-Harada (VKH) disease is chronic, bilateral, granulomatous panuveitis characterized by exudative retinal detachments associated with poliosis, vitiligo, alopecia, and central nervous system and auditory signs [1]. The exact cause of VKH disease remains unknown, but evidence suggests that it involves a T-lymphocyte-mediated autoimmune process directed against one or more antigens found on or associated with melanocytes. Several studies demonstrated that tyrosinase family proteins are the antigens specific to VKH disease [2–5] and that VKH disease is characterized by a T helper type 1 cell-mediated immune response [6, 7].

Vision-threatening complications have been clearly recognized to occur in the chronic, recurrent phase of VKH disease, namely, cataract, glaucoma, subretinal neovascular membranes, and subretinal fibrosis. The occurrence of these complications is known to be associated with a worse visual outcome [1, 8, 9]. The principles of therapy of VKH disease are to suppress the initial intraocular inflammation with early and aggressive use of systemic corticosteroids, followed by slow tapering [1, 10]. The addition of conventional immunosuppressive agents to systemic corticosteroids has been advocated [11, 12]. Such combined treatment may shorten the duration of the disease, may prevent progression into the chronic stage, and may reduce the incidence of extraocular manifestations and complications as well.

Khairallah et al. [13] described the first case of RPE proliferation leading to subretinal fibrosis in a patient with Vogt-Koyanagi-Harada disease. To the best our knowledge, our case represents the second reported case of RPE proliferation leading to subretinal fibrosis in chronic VKH disease. Our case is unique in terms of the exuberant pseudotumoral retinal RPE proliferation in multiple locations at the juxtapapillary area and periphery.

## 2. Case Report

**Figure 1:**
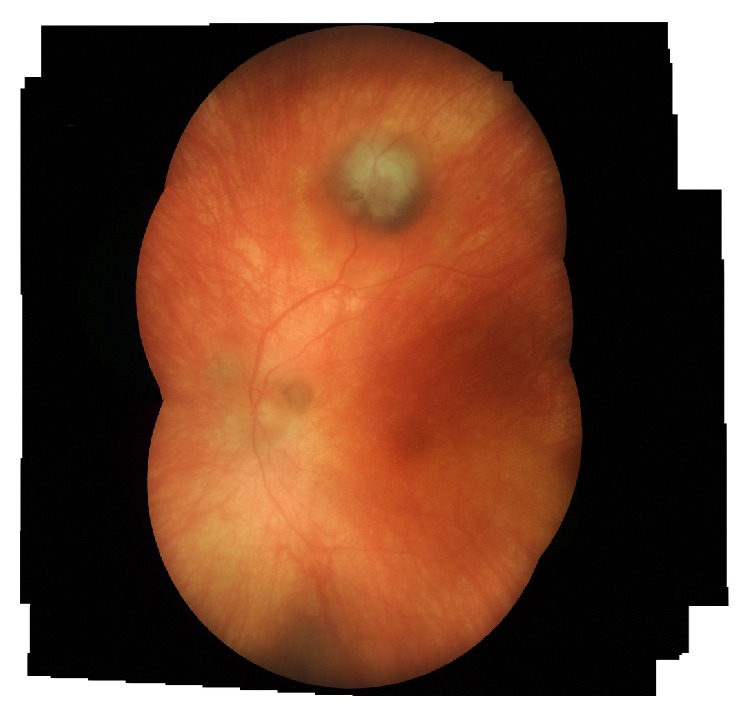
(A-B) Fundus examination of LE showed features of chronic recurrent Vogt-Koyanagi-Harada disease, including “sunset glow fundus,” hyperpigmented striae, and multiple atrophic chorioretinal spots in the periphery (not shown); darkly pigmented exuberant subretinal mass extended to the periphery with associated subretinal fibrosis. There were 2 smaller pigmented subretinal lesions located around the optic disc.

**Figure 2:**
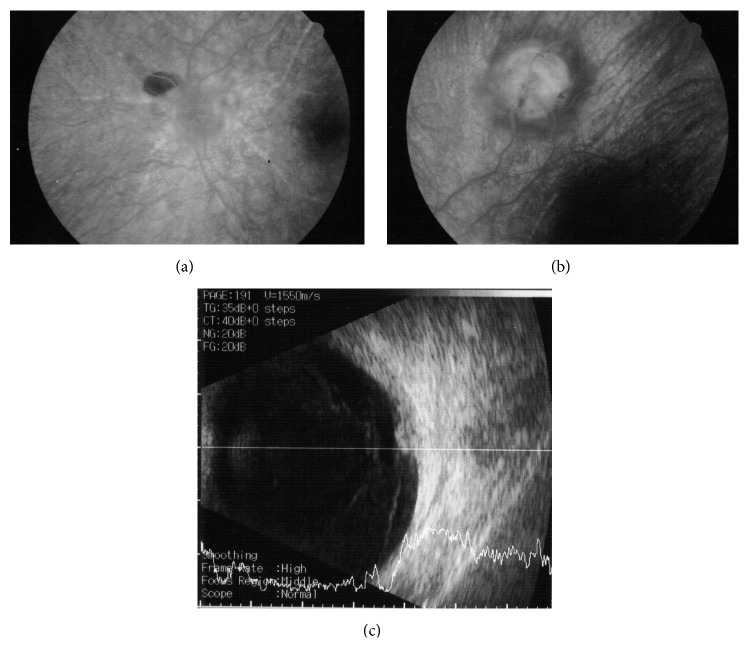
(a-b) Fluorescein angiography (FA) demonstrated background hyperfluorescence corresponding to the “sunset glow fundus” and hyper- and hypofluorescence of the RPE proliferating lesions. (c) A B-scan ultrasound of the larger peripheral RPE proliferations showed a dome-shaped mass 2 mm in height, high internal reflectivity, and a partial posterior vitreous detachment.

**Figure 3:**
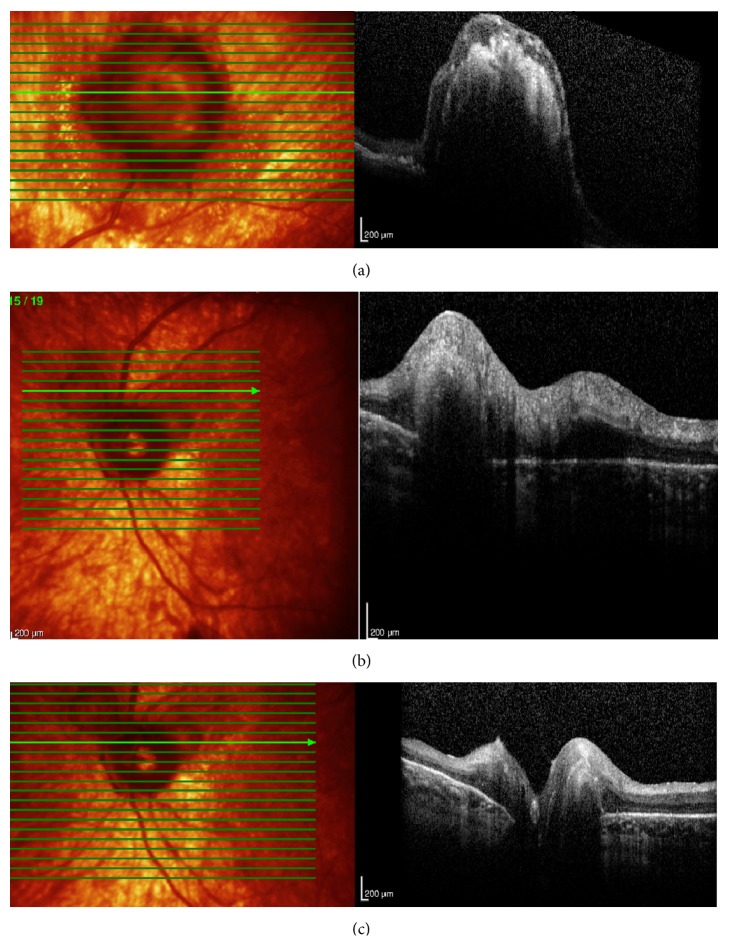
(a–c) Optical coherent tomography (OCT) in LE showed 2 lesions around the optic disc and 1 on the temporal vascular superior arcade. Overlying the dome-shaped elevation of the outer retinal layers, the choroid was thickened and the retinal pigment epithelium (RPE) was irregular.

A 50-year-old woman with a 20-year history of panuveitis associated with exudative retinal detachment in the left eye (LE) that had been treated with long-term oral corticosteroids was referred to our center. She had a juxtapapillary and peripheral subretinal hyperpigmented mass in LE. Her right eye had history of a chronic rhegmatogenous retinal detachment after complicated cataract surgery 5 years before presentation and no light perception visual acuity. Her visual acuity was 20/400 OS. Slit-lamp examination showed periocular vitiligo, poliosis, and posterior synechiae. There were no cells and flare in the anterior chamber or vitreous haze. Intraocular pressure was 12 mm Hg OU. Fundus examination of LE showed features of chronic recurrent VKH disease, including “sunset glow fundus,” hyperpigmented striae, and multiple atrophic chorioretinal spots in the periphery; a darkly pigmented exuberant subretinal mass extended to the periphery with associated subretinal fibrosis. There were 2 smaller pigmented subretinal lesions located around the optic disc (Figure 1). Fluorescein angiography (FA) demonstrated background hyperfluorescence corresponding to the “sunset glow fundus” and hyper- and hypofluorescence of the RPE proliferating lesions (Figures 2(a)-2(b)). A B-scan ultrasound of the larger peripheral RPE proliferations showed a dome-shaped mass 2 mm in height, high internal reflectivity, and a partial posterior vitreous detachment (Figure 2(c)). Optical coherent tomography (OCT) showed 2 lesions around the optic disc and 1 on the temporal vascular superior arcade. Overlying the dome-shaped elevation of the outer retinal layers, the choroid was thickened and the retinal pigment epithelium (RPE) was irregular (Figure 3).

## 3. Discussion

Early and aggressive high-dose systemic corticosteroid therapy has become the mainstay therapy of VKH disease. Patients with VKH disease adequately treated with corticosteroids have a favourable visual prognosis. However, the addition of conventional immunosuppressive agents to systemic corticosteroids has been advocated to prevent the progression of VKH disease, lessen its duration, prevent severe ocular complications, and prevent systemic diseases such as ear, skin, or hair lesions [11, 12]. Our case exemplifies that complications may occur in patients with VKH disease if they are not properly treated.

The present case shows that RPE proliferation leading to subretinal fibrosis may occur in patients with VKH disease. The first case was described by Khairallah et al. in 2006 [13] who demonstrated that the fibrotic mass can be covered by pigmented cells. To the best of our knowledge, our case is the second described pseudotumoral RPE proliferation and adds multiple lesions including one in the peripheral retina. Although this complication has rarely been reported, a high index of suspicion is warranted for early diagnosis and avoids unnecessary treatment of a pseudotumor. In addition, we have to keep in mind the possibility that other chronic inflammatory conditions could potentially be associated with RPE proliferation and pseudotumors.
